# A Combined Activity of Thrombin and P-Selectin Is Essential for Platelet Activation by Pancreatic Cancer Cells

**DOI:** 10.3390/ijms22073323

**Published:** 2021-03-24

**Authors:** Reza Haschemi, Lukas Maria Gockel, Gerd Bendas, Martin Schlesinger

**Affiliations:** Pharmaceutical Institute, University of Bonn, An der Immenburg 4, 53121 Bonn, Germany; Reza.Haschemi@uni-bonn.de (R.H.); Lukas.Gockel@uni-bonn.de (L.M.G.); gbendas@uni-bonn.de (G.B.)

**Keywords:** dense granule release, hypercoagulability, platelets, pancreatic cancer, platelet aggregation, P-selectin, thrombin, tissue factor

## Abstract

Pancreatic cancer patients have an elevated risk of suffering from venous thrombosis. Among several risk factors that contribute to hypercoagulability of this malignancy, platelets possess a key role in the initiation of clot formation. Although single mechanisms of platelet activation are well-known in principle, combinations thereof and their potential synergy to mediate platelet activation is, in the case of pancreatic cancer, far from being clear. Applying an inhibitor screening approach using light transmission aggregometry, dense granule release, and thrombin formation assays, we provide evidence that a combination of tissue factor-induced thrombin formation by cancer cells and their platelet P-selectin binding is responsible for AsPC-1 and Capan-2 pancreatic cancer cell-mediated platelet activation. While the blockade of one of these pathways leads to a pronounced inhibition of platelet aggregation and dense granule release, the simultaneous blockade of both pathways is inevitable to prevent platelet aggregation completely and minimize ATP release. In contrast, MIA PaCa-2 pancreatic cancer cells express reduced levels of tissue factor and P-selectin ligands and thus turn out to be poor platelet activators. Consequently, a simultaneous blockade of thrombin and P-selectin binding seems to be a powerful approach, as mediated by heparin to crucially reduce the hypercoagulable state of pancreatic cancer patients.

## 1. Introduction

Among all common human malignancies, patients suffering from pancreatic cancer have the highest risk of clinically apparent venous thromboembolism in the first year after cancer diagnosis [1,2]. From a clinical perspective, several risk factors have been identified to be associated with hypercoagulability, classified as patient-, tumor- or treatment-related factors. In the case of pancreatic cancer, the tumor localization and stage of the disease, a probable metastatic burden, mucin secretion by the cancer cells, chemotherapeutic treatment, surgical interventions, or obesity, among other factors can shift the hemostatic equilibrium to a pre- or even thrombotic state [3,4,5]. While the clinical observations and identified risk factors are relatively clear and unambiguous, the molecular mechanisms contributing to a status of increased coagulation are only partially understood.

Platelet aggregation induced by cancer cells is regarded as a key factor and initiator of the hypercoagulable state in malignancies. For instance, platelets are involved in the release of neutrophil extracellular traps that activate coagulation factor XII (Hageman factor) and subsequently the intrinsic coagulation cascade [6,7,8,9]. The close interaction between tumor cells and platelets also implies many advantages for cancer progression and metastasis. Activated platelets secrete a vast number of different chemokines from their granules which instigate a modulation of the local immune response and mitigate the apoptosis-inducing capacity of T- and NK-cell attacks [10,11,12]. Furthermore, platelets also induce a transition of the tumor cell phenotype from an epithelial to mesenchymal (EMT) one, which confers stem cell-like properties to the tumor cells associated with increased motility and invasiveness [13]. In fact, platelets contribute to cancer progression by numerous mechanisms and have a pivotal impact on the protumorigenic capacity.

Consequently, efficient inhibition of platelet activity could comprise a reduction of the hypercoagulable state and the associated ischemic events of cancer patients and could additionally constrain the tumor cell platelet interaction. For pancreatic cancer as well as other cancer entities, several mechanisms contributing to platelet activation have been described [4,14]. The greatest significance in hypercoagulability and platelet aggregation is related to the tissue factor (TF) expression on pancreatic cancer cells. TF activates the plasmatic coagulation cascade, which culminates in thrombin formation, fibrin generation, and finally platelet activation by protease-activated receptor-1 (PAR-1) cleavage [15,16,17]. Furthermore, some tumor cells secrete soluble mediators like ADP [18], or thromboxane A2 (TXA2), which also affect the status of platelet activation by binding to platelet purinergic receptors P2Y_12_, P2Y_1_, and P2X_1_, or TXA2 receptor, respectively [19].

A further line of evidence suggests a platelet activation mediated by direct contact between tumor cells and platelets involving different platelet receptors. For instance, platelet glycoprotein VI and integrin α_6_β_1_ have been identified to induce platelet activation upon binding to tumor cells expressing galectin-3 or ADAM-9, respectively [20,21]. Podoplanin expressed by stromal fibroblasts in the pancreatic tumor tissue is supposed to induce platelet activation via the C-type lectin-like receptor-2 (CLEC-2) [22]. Platelet P-selectin, traditionally regarded as a simple adhesion receptor is also capable of expediting platelet aggregation and secretion [23,24]. For pancreatic cancer cells, selective platelet activation mechanisms have been investigated individually, but data of combinatorial approaches, blocking different pathways concomitantly to confine platelet activation are hardly available.

In the present study we elucidate the mode of action and differences for pancreatic cancer cells to induce platelet activation. While MIA PaCa-2 cells turn out to be generally weak platelet activators, AsPC-1 and Capan-2 pancreatic cancer cells utilize preferentially two different pathways for platelet activation in parallel. First, tumor cell-expressed TF activates the coagulation cascade triggering finally PAR-1 on platelets. Furthermore, a P-selectin mediated direct interaction between AsPC-1 and Capan-2 cancer cells, respectively, and platelets contributes crucially to platelet aggregation and secretion. Thus, a concomitant blockade of thrombin and P-selectin completely prevents platelet activation and appears to be a promising approach for the reduction of hypercoagulability and interference in the platelet tumor cell communication.

## 2. Results

### 2.1. AsPC-1 and Capan-2 Cells Induce Human Platelet Aggregation and ATP Secretion

**Figure 1:**
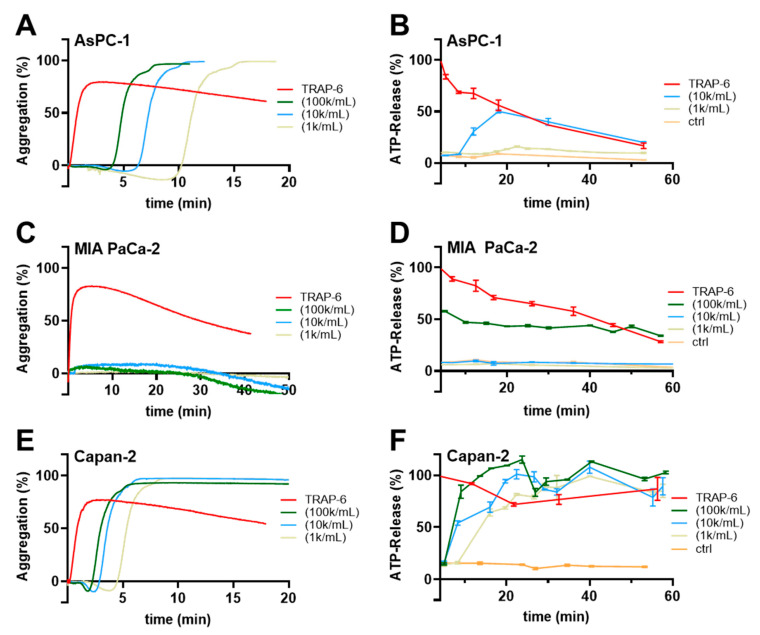
Pancreatic cancer cell induced human platelet aggregation and ATP secretion. Representative traces showing platelet aggregation in response to increasing concentrations of AsPC-1 (**A**), MIA PaCa-2 (**C**), Capan-2 (**E**) pancreatic cancer cells, or TRAP-6, respectively, for 20–50 min (*n* = 5). (**B**) Quantification of ATP release from resting platelets, platelets activated with TRAP-6, or co-incubated with AsPC-1 (**B**), MIA PaCa-2 (**D**), Capan-2 (**F**) pancreatic cancer cells for 60 min using a luciferin-based ATP-determination kit (*n* = 5).

To gain insight into the mechanisms by which pancreatic cancer cells commonly promote platelet activation, at first, we tested different concentrations of AsPC-1, MIA PaCa-2 and Capan-2 pancreatic cancer cells for their abilities to induce a platelet aggregation, and ATP secretion from platelet-dense granules. For AsPC-1 cells, concentrations ranging from 1 × 10^3^ to 1 × 10^5^ cells/mL triggered a platelet aggregation after approximately 4 min (Figure 1A). ATP release from platelets started 10 min after AsPC-1 cells had been added with a maximum release after 20 min (Figure 1B). Application of the PAR-1 agonist TRAP-6 induced an immediate ATP release, whereas platelets alone exhibited hardly any ATP secretion. Thus, in the following experiments, ATP quantification was conducted 20 min after tumor cell administration. In contrast to AsPC-1 cells, MIA PaCa-2 cells failed to induce a platelet aggregation within 50 min even at a concentration of 1 × 10^5^ cells/mL (Figure 1C). In the subsequent ATP release quantification, an application of 1 × 10^5^ MIA PaCa-2 cells/mL exhibited an ATP concentration of 50% compared to TRAP-6 activated platelets which was potentially derived from MIA PaCa-2 cells. Lower numbers of cells had no effect on the ATP levels (Figure 1D). Thus, MIA PaCa-2 cells are obviously poor platelet activators compared to AsPC-1 cells. The third pancreatic cancer cell line Capan-2 revealed as the most potent platelet activator and induced platelet aggregation after 2 min for 1 × 10^5^ cells/mL (Figure 1E). Application of 1 × 10^3^ cells/mL to the platelets even mediated a start of platelet aggregation after 5 min whereas 1 × 10^3^ AsPC-1 cells took 10 min for platelet aggregation. ATP secretion from platelets started 10 min after addition of 1 × 10^3^ Capan-2 cells/mL and reached a maximum after 30 min. Higher concentrations of Capan-2 cells induced a platelet ATP secretion only 5 min after addition.

### 2.2. Thrombin, Factor Xa, P-Selectin, Integrin GPIIbIIIa, and P2Y_12_ Receptor Contribute to AsPC-1 Mediated Platelet Activation

**Figure 2:**
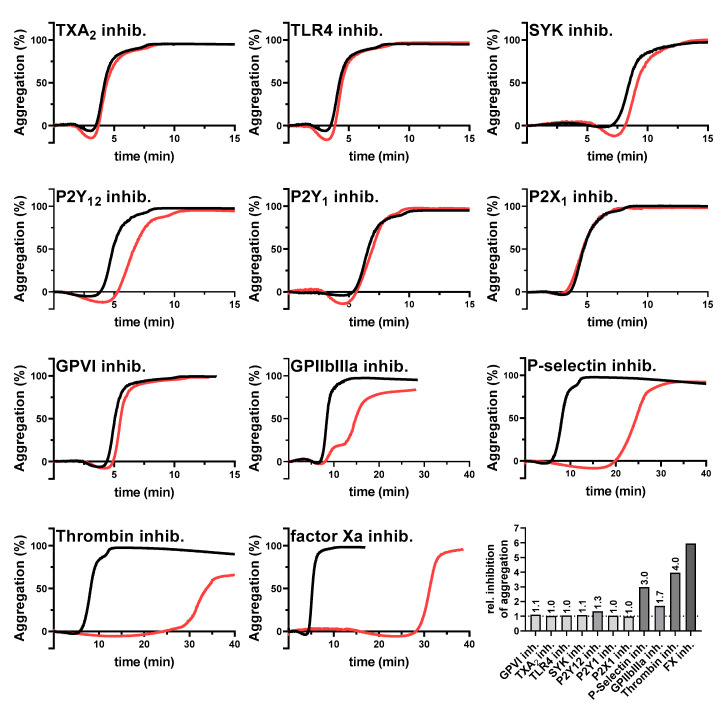
Inhibition of AsPC-1 cell-mediated platelet aggregation. Representative aggregation curves of platelets activated with 1×10^4^ AsPC-1 cells/mL (black curves), or platelets preincubated with inhibitors for TXA2 receptor, TLR4, SYK tyrosine kinase, purinergic receptors P2Y_12_, P2Y_1_, P2X_1_, GPVI, GPIIbIIIa, P-selectin, thrombin, or factor Xa, respectively (red curves) (*n* = 5). Effects of the different inhibitors on platelet aggregation are shown as ratio of time to half-maximal aggregation of treated versus untreated platelets.

To identify which platelet activation pathways are triggered by the tumor cells, we selected the AsPC-1 cells for a screening with different potential platelet inhibitors. Platelet aggregation was initiated by 1 × 10^4^ AsPC-1 cells/mL as control while platelets were preincubated with certain inhibitors in the indicated experiments (Figure 2). However, AsPC-1 mediated aggregation of platelets displayed a slight deviation in the on-set time, which might potentially be caused by donor-specific variations. Nevertheless, since platelets were tested for full functionality, these deviations were not further considered.

Notably, blockade of the TXA2 receptor, toll-like receptor 4, GPVI, purinergic receptors P2Y_1_, P2X_1_, or the intracellular spleen tyrosine kinase (SYK) had no impact on AsPC-1 induced aggregation. Since the SYK kinase is a downstream mediator for the platelet receptors GPI-IX-V, FcγRIIA, CLEC-2, or GPVI, it is likely that these receptors do not contribute to a AsPC-1 mediated platelet activation. Supportive of this finding, a GPVI inhibition had no effect on platelet aggregation. In contrast, a blockade of integrin GPIIbIIIa as well as P2Y_12_ receptor delayed AsPC-1 induced platelet aggregation partially. Since autocrine secreted ADP amplifies platelet activation by P2Y_12,_ and integrin GPIIbIIIa crosslinks platelets via fibrinogen initiating an “outside-in signaling”, it is conceivable that a blockade of both receptors had an impact on the late phase of platelet aggregation. Furthermore, application of a pan-selectin antagonist (Bimosiamose, which blocks P-, E- and L-selectin) induced a strong delay in platelet aggregation. Similar results were obtained for a selective thrombin (Argatroban), and factor Xa inhibitor (Rivaroxaban), respectively. Aggregation started 30 min after AsPC-1 cell administration with both inhibitors. In comparison, untreated platelets started to aggregate 5–10 min after tumor cell addition. Furthermore, for a better comparability of the single aggregation experiments, the ratio of time to half-maximal aggregation of treated versus untreated platelets was calculated (Figure 2).

**Figure 3:**
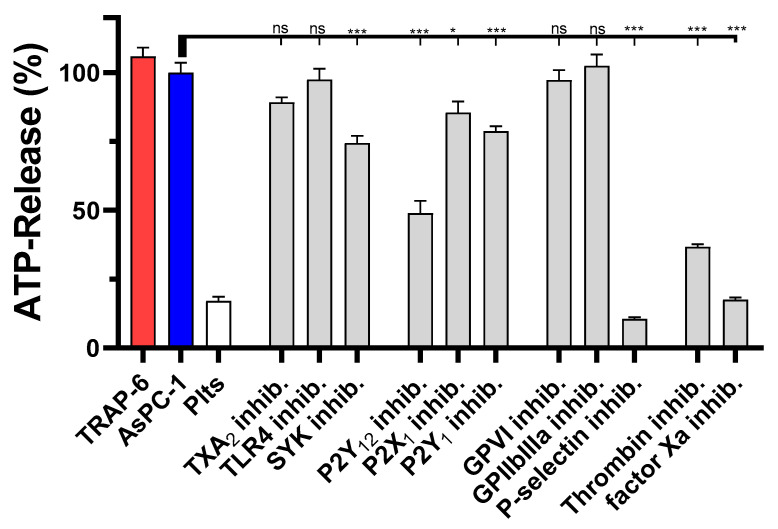
Inhibition of AsPC-1 cell induced platelet dense granule secretion. Quantification of ATP release from resting platelets, platelets co-incubated with 1 × 10^4^ AsPC-1 cells, or platelets activated with TRAP-6, respectively, after 20 min. In some experiments platelets were preincubated with inhibitors for TXA2 receptor, TLR4, SYK tyrosine kinase, purinergic receptors P2Y_12_, P2X_1,_ P2Y_1_, GPVI, GPIIbIIIa, P-selectin, thrombin, or factor Xa, respectively, before the addition of AsPC-1 cells. Data are means of *n* = 3–5 (±SD), asterisks indicate statistical significance: * *p* < 0.05; *** *p* < 0.001.

We next tested the impact of the different inhibitors on the platelet-derived ATP secretion. Blockade of SYK kinase, or purinergic receptors P2Y_12_, P2Y_1_, or P2X_1_ had only a minor impact on ATP secretion, whereas a thrombin or P-selectin inhibition had the strongest effects to attenuate platelet-derived ATP release (Figure 3). For P-selectin inhibition, ATP levels were reduced by 90%, and in the case of thrombin inhibition by Argatroban by 60% compared to untreated AsPC-1 activated platelets. Thus, the results obtained in the platelet aggregation experiments were corroborated by the dense granule release quantification.

### 2.3. Expression of TF and P-Selectin Ligands on Pancreatic Cancer Cells

**Figure 4:**
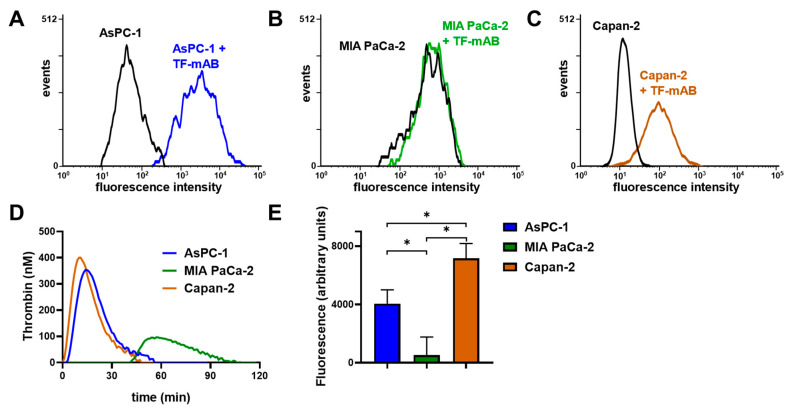
TF and P-selectin ligand determination on AsPC-1, Capan-2, and MIA PaCa-2 cells. Quantification of TF expression by flow cytometry on (**A**) AsPC-1, (**B**) MIA PaCa-2, and (**C**) Capan-2 cells. AsPC-1 and Capan-2 cells exhibited a pronounced TF expression whereas MIA PaCa-2 cells revealed hardly any expression. (**D**) Thrombin generation of AsPC-1, Capan-2, and MIA PaCa-2 cells was determined in platelet-rich-plasma without recalcification. (**E**) Quantification of P-selectin ligand expression was determined with a P-selectin adhesion assay for AsPC-1, Capan-2, and MIA PaCa-2 cells. AsPC-1 and Capan-2 exhibited an enhanced binding to immobilized, recombinant human P-selectin compared to MIA PaCa-2 cells. Figures (**A**–**D**) illustrate representative data of at least five identical experiments. Data of the static adhesion assay (**E**) are means of at least *n* = 5 (±SD), asterisks indicate statistical significance: * *p* < 0.05.

To confirm whether the coagulation cascade and P-selectin signaling are potentially involved in AsPC-1 and Capan-2 induced platelet activation, we determined TF expression on the respective cell lines by flow cytometry and compared these with MIA PaCa-2 cells. AsPC-1 and Capan-2 cells expressed a high level of TF on the cell membrane, whereas MIA PaCa-2 cells exhibited hardly any TF expression (Figure 4A–C). To substantiate the relevance of TF expression as a trigger for downstream thrombin generation, we followed that in a suitable assay and found a fast thrombin formation induced by AsPC-1 and Capan-2 cells (5 min after tumor cell addition) (Figure 4D). In contrast, MIA PaCa-2 cells revealed a slow thrombin formation that started 40 min after tumor cell addition.

Next, we focused on the expression of P-selectin ligands by the indicated tumor cells with a P-selectin adhesion assay. P-selectin was traditionally regarded as a simple adhesion receptor on platelets that contributed to aggregate formation and stability without any signaling function [25,26,27]. Nevertheless, accumulating evidence suggests that P-selectin indeed has a signaling function that regulates the status of platelet activity [23,24]. AsPC-1 and Capan-2 cells revealed an increased binding to immobilized P-selectin compared to MIA PaCa-2 cells, which refers to an enhanced expression of P-selectin ligands on membranes of both cell lines (Figure 4E).

These results could serve as an explanation for the inability of MIA PaCa-2 cells to induce platelet aggregation and secretion (Figure 1C,D), since these cells solely express basal TF levels and less P-selectin ligands.

### 2.4. Combinatory Inhibition of Thrombin and P-Selectin

**Figure 5:**
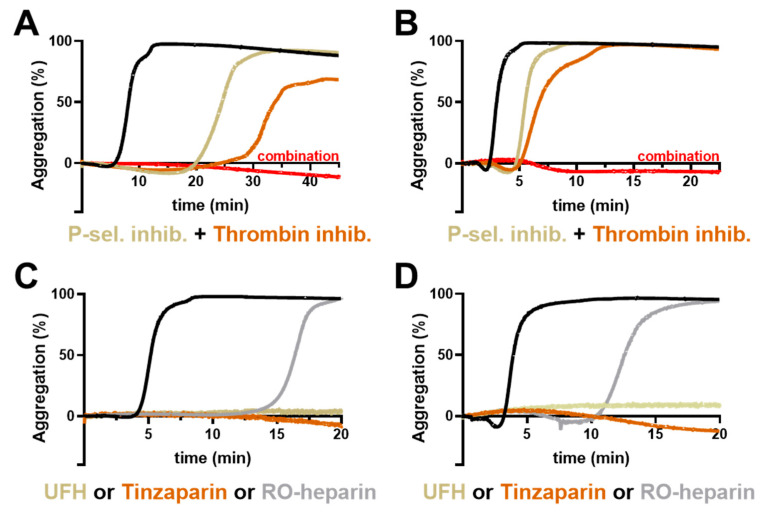
Impact of inhibitor combinations on platelet aggregation. Representative aggregation curves of platelets activated with 1 × 10^4^ AsPC-1 (**A**) or Capan-2 (**B**) cells/mL, (black curves). In some experiments, platelets were preincubated with thrombin inhibitor Argatroban, a P-selectin inhibitor (Bimosiamose), or combination of both inhibitors, (*n* = 5). Representative aggregation curves of platelets activated with 1 × 10^4^ AsPC-1 (**C**) or Capan-2 (**D**) cells/mL (black curves). In some experiments, platelets were preincubated with unfractionated heparin, Tinzaparin, or RO-heparin, (*n* = 5).

The above data indicate that thrombin generation and P-selectin contribute individually to platelet activation and their inhibition attenuates platelet activity to a distinct degree. Consequently, we investigated whether a combinatorial approach using these different platelet inhibitors is an option to further suppress platelet activation induced by AsPC-1 or Capan-2 cells, and to obtain an insight as to whether they act in synergy or in addition. Therefore, inhibitor combinations were added to the platelets before aggregation was induced. A combined inhibition of P-selectin (Bimosiamose) and thrombin (Argatroban) completely blocked aggregation for at least 60 min for AsPC-1 cells (Figure 5A) and Capan-2 cells (Figure 5B). Thus, a combination of P-selectin blockade and inhibition of the plasmatic coagulation cascade seems likely for synergistic activity in attenuating platelet aggregation by both tumor cell lines.

To validate this hypothesis, we applied heparin derivatives in the next experiments since heparin blocks thrombin/factor Xa and has also been revealed as a potent P-selectin inhibitor [28,29,30]. The application of unfractionated heparin or low-molecular-weight heparin Tinzaparin completely blocked platelet aggregation for 60 min (Figure 5C,D) and corroborated our previous assumption of a two-pronged platelet activation mechanism consisting of P-selectin and thrombin generation induced by AsPC-1 and Capan-2 pancreatic cancer cells. In order to differentiate the potential antithrombotic and P-selectin inhibitory potential of heparin, we applied a non-anticoagulant reduced oxyheparin (RO-heparin), which is a potent P-selectin inhibitor. Notably, RO-heparin delayed platelet aggregation, but failed to block aggregation completely, which further confirmed the combined role of P-selectin and thrombin for platelet activation (Figure 5C,D). In contrast the same concentration of unfractionated heparin thoroughly prevented platelet aggregation.

Together, these findings indicate that TF expression on pancreatic cancer cells and the subsequent generation of thrombin contribute to a procoagulant cellular phenotype. Furthermore, P-selectin ligand expression on tumor cells potentially contributes to platelet interaction and finally platelet activation.

## 3. Discussion

The close interaction between tumor cells and platelets is well-known and for pancreatic cancer patients a highly relevant fact that determines their coagulation status. Some of the signaling pathways that account for pancreatic cancer cell-mediated platelet activation have been identified, whereas others are completely unexplored [4]. In particular, approaches combining different inhibitory strategies could be conducive to elucidate signaling pathways that cooperate in platelet activation.

In this work, we show that TF and P-selectin ligand expression on pancreatic cancer cells potentially work in concert for platelet aggregation and secretion.

For this purpose, we utilized three different pancreatic cancer cell lines (AsPC-1, Capan-2, and MIA PaCa-2) to analyze their procoagulant properties. At first, the focus was directed on TF expression on the tumor cell lines as a relevant inductor of the plasmatic coagulation cascade. Capan-2 and AsPC-1 cells displayed high levels of TF and induced thrombin formation, platelet aggregation, and secretion. Blockade of the coagulation cascade (thrombin or factor Xa) led to a delay in aggregation and reduced ATP secretion. Similar results were obtained by blockade of platelet P-selectin with the pan-selectin inhibitor Bimosiamose. The combination of both inhibitors finally prevented platelet aggregation and ATP release completely. In contrast, MIA PaCa-2 cells exhibited hardly any TF expression and also P-selectin ligand expression was relatively low compared to AsPC-1 and Capan-2 cells. Those cells were almost unable to initiate proper platelet activation.

TF is normally expressed on subendothelial vascular smooth muscle cells and exposed to the blood flow upon vascular endothelial cell disruption [31]. Besides coagulation, TF has a broad implication in cancer progression and angiogenesis. Membrane-bound TF can form a complex with the integrins α_3_β_1_ or α_6_β_1_ and factor VIIa which exerts its cellular effects by PAR-2 activation. This leads to MAP or PI3 kinase activation, cell survival, cytoskeletal rearrangements, and secretion of pro-angiogenic factors, such as vascular endothelial growth factor, CXCL-1, and IL-8 among others [16,32,33]. From a clinical point of view, TF expression also correlated with the histological grade of the tumor, in particular, immunohistochemical analysis revealed that most of the poorly differentiated pancreatic tumors expressed TF whereas well-differentiated tumors had a lower expression rate, indicating an association between more aggressive histological subtypes with TF expression [34]. Thus, for pancreatic cancer, TF is a potential biomarker and relevant target concerning hypercoagulability and tumor progression.

Furthermore, the direct juxtacrine contact between platelets and pancreatic cancer cells also contributes to platelet activation. Obviously, in this process, P-selectin takes a key role. Platelet P-selectin has been well-known for decades for mediating the interaction of different tumor cells with platelets [28,29,35,36]. Nevertheless, P-selectin was always regarded as a simple adhesion receptor without any signaling function whereas recent reports suppose that P-selectin does participate in platelet activation. The first evidence came from P-selectin deficient mice who had a 40% increased bleeding time in comparison to wild type mice upon tail tip amputation [37]. Additionally, the supernatant of P-selectin deficient platelets cocultured with B16 melanoma cells revealed decreased concentrations of vascular endothelial growth factor (VEGF). This finding validates that P-selectin has an impact on platelet secretion and confirms our data on platelet ATP release [38]. Furthermore, antibody-mediated crosslinking of P-selectin on human platelets induced a calcium entry [39], and stimulation of platelets with thrombin, phorbol 12-myristate 13-acetate, or collagen, respectively, culminated in histidine, serine and threonine phosphorylation of P-selectin cytoplasmic residues [40,41]. Becker et al. revealed that P-selectin and p38 MAP kinase signaling regulate the secretion of acid sphingomyelinase from platelet granules after melanoma cell-mediated activation [42]. P-selectin and acid sphingomyelinase secretion contributed to melanoma cell adhesion and finally pulmonary tumor metastasis. Wang and colleagues recently described crosstalk between P-selectin, the cytoskeletal protein talin-1 and integrin GPIIb/IIIa [43]. P-selectin recruited talin-1 to its cytoplasmic tail and this led to an activation of integrin GPIIb/IIIa, an infiltration of platelets into insulinoma or colon cancer tissue. Furthermore, we recently demonstrated that P-selectin contributes to platelet activation mediated by breast cancer cells involving Src-family kinases Fyn, and Hck, protein kinase B (Akt), and extracellular signal-regulated kinase (Erk) [24]. P-selectin blockade reduced platelet aggregation and modulated platelet secretome. Thus, platelet P-selectin seems to provide a comprehensive function in platelets including adhesion, intracellular signaling, and receptor crosstalk.

Our data support the notion that directly acting oral anticoagulants like Rivaroxaban or Argatroban can prevent platelet activation and a hypercoagulable state, but fail to block platelet activation by direct contact formation with pancreatic cancer cells. Thus, heparin which blocks both the plasmatic coagulation cascade and simultaneously P-selectin could be beneficial for the anticoagulant treatment of pancreatic cancer patients [44,45,46]. Nevertheless, analysis of further pancreatic cancer cell lines could reveal additional combinations of mechanisms that contribute to platelet activation that are beyond the present study. Moreover, the in vivo situation is much more complex compared to the in vitro experiments and includes further cell entities, e.g., endothelial cells and different leukocyte subpopulations, which also have a tremendous impact on platelet activity and coagulation.

Thus, a considerable amount of work remains to fully comprehend which signaling pathways generally work in concert to orchestrate platelet reactivity and the associated coagulation status in pancreatic cancer patients. Nevertheless, the combination of TF and P-selectin seems to be at least partially responsible for coagulability of pancreatic cancer cells. However, our data emphasize the role of heparin since this drug combines antithrombotic and antiadhesive properties in a unique manner, which could be a further argument for the still ongoing debate whether and how heparin possesses further antitumor activities in a clinical application.

## 4. Materials and Methods

### 4.1. Cell Culture

Human AsPC-1 and MIA PaCa-2 pancreas carcinoma cells were obtained from Prof. Dr. Ulrich Massing, University Hospital Freiburg, Germany, Clinic for Tumor Biology and their identity was confirmed by Short-tandem repeat profiling. AsPC-1 cells were grown in RPMI 1640 medium (PAN Biotech, Aidenbach, Germany) containing 10% (*v/v*) fetal calf serum (FCS, Sigma Aldrich, Steinheim, Germany), 100 U/mL penicillin, and 100 µg/mL streptomycin (PAN Biotech). MIA PaCa-2 cells were grown in Dulbecco’s Modified Eagle’s Medium (DMEM) (PAN Biotech, Aidenbach, Germany) containing 10% (*v/v*) fetal calf serum (FCS, Sigma Aldrich, Steinheim, Germany), 100 U/mL penicillin, and 100 µg/mL streptomycin (PAN Biotech). Capan-2 cells were kindly obtained from Prof. Ingo Schmidt-Wolf, University Hospital Bonn, Germany, and cultivated in RPMI 1640 medium (PAN Biotech) containing 10% (*v/v*) fetal calf serum (FCS, Sigma Aldrich), 100 U/mL penicillin and 100 µg/mL streptomycin (PAN Biotech). All cell lines were incubated at 37 °C in a humidified atmosphere containing 5% (*v/v*) CO_2_.

For subcultivation, cell lines were treated with trypsin/EDTA (5 g/L trypsin; 0.2 g/L EDTA × 4 Na, Sigma Aldrich) for 5 min at 37 °C. Mycoplasma check was routinely performed every month and cell identity was validated using a STR profile analysis.

### 4.2. Platelet Isolation and Activation

Platelet-rich-plasma from different donors was obtained from the Institute for Experimental Hematology and Transfusion Medicine, Medical Centre, University of Bonn, in accordance with the declaration of Helsinki. Isolated human platelets (Plts) in buffer were prepared from platelet-rich-plasma by centrifugation (670 g, 10 min, 22 °C) and resuspension to a concentration of 4 × 10^8^ Plts/mL in recalcified (1 mM) platelet buffer (10 mM HEPES, 137 mM NaCl, 2.6 mM KCl, 1 mM MgCl_2_, 13.8 mM NaHCO_3_, 0.36 mM NaH_2_PO_4_, 5.5 mM d-glucose). Before use, 1% platelet-poor-plasma was added. In some experiments, prior to activation, platelets were preincubated with inhibitors against GPVI (Losartan, 20 µM, Biotechne, Wiesbaden-Nordenstadt, Germany), TXA2 (Seratrodast, 10 µg/mL, MedChem Express, Hycultec GmbH, Beutelsbach, Germany), TLR4 (TAK-242, 5 µM, MedChem Express), Syk (BAY61-3606, 10 µM, Santa Cruz Biotechnology, Heidelberg, Germany), GPIIbIIIa (Eptifibatide, 10 µg/mL, MedChem Express), purinergic receptors P2Y_12_ (Ticagrelor, 1 µM), P2Y_1_ (BPTU, 10 µM), P2X_1_ (PSB-18164, 10 µM) (were kindly provided by Prof. Dr. Christa E. Müller, Pharmaceutical Institute, University of Bonn), factor Xa (Rivaroxaban, 0.5 nM, MedChem Express), and thrombin (Argatroban, 10 µM, MedChem Express), respectively. RO-heparin was kindly provided by Dr. Giangiacomo Torri, Ronzoni Institute for Chemical and Biochemical Research, Milan, Italy, and applied at a concentration of 5 µg/mL. LMWH Tinzaparin (Innohep^®^, LEO Pharma, Neu-Isenburg, Germany) and UFH (Heparin-Natrium-25000-ratiopharm, Ratiopharm, Ulm, Germany) were used at a final concentration of 1 international unit (IU)/mL.

For P-selectin blockade, Bimosiamose, which is a pan-selectin antagonist (TBC-1269; 1,6-bis(3-(3-carboxymethylphenyl)-4-(2-alpha-d-mannopyranosyloxy)phenyl)hexane (100 µg/mL; formerly Revotar Biopharmaceuticals, Hennigsdorf, Germany), was added to the platelets in buffer for 30 min. TLR4 antagonist TAK-242, GPVI inhibitor Losartan, Syk inhibitor BAY61-3606, TXA2 inhibitor Seratrodast, factor Xa inhibitor Rivaroxaban, thrombin inhibitor Argatroban, P2Y_12_ inhibitor Ticagrelor, P-selectin inhibitor Bimosiamose, P2X_1_ inhibitor PSB-18164, and P2Y_1_ inhibitor BPTU were solved and diluted in DMSO. In the experiments, a final DMSO concentration of 0.5% was not exceeded and pure DMSO was also added in the corresponding control experiments to detect potential DMSO related effects.

Prior to each experiment, aggregation and secretion capacities of the platelets were checked by light transmission aggregometry and platelet dense granule secretion assays to exclude donor specific differences.

### 4.3. Flow Cytometric Detection of TF Expression

TF expression on AsPC-1, Capan-2, and MIA PaCa-2 pancreatic cancer cells was investigated by flow cytometry. Briefly, cells were detached using an EDTA/DPBS solution and after centrifugation, the pellet was suspended in blocking buffer (3% BSA in DPBS) to a concentration of 1000 cells/µL and blocked for 15 min. Then, the wells were washed twice using a wash buffer (0.5% BSA, 0.1% sodium azide, DPBS). In order to label TF, cells were incubated for 45 min with a primary anti-TF antibody (Clone #323519, R&D systems, Wiesbaden Nordenstadt, Germany) at a concentration of 2.5 µg/10^6^ cells. Subsequently, the samples were washed twice and incubated for 35 min with a FITC-conjugated, secondary antibody using 0.75 µg/10^6^ cells (Santa Cruz Biotechnology Inc., Santa Cruz, CA, USA). For each sample 1 × 10^4^ cells were analyzed with a Guava easyCyte 11 HT reader (Merck Millipore, Billerica, MA, USA) after washing twice with DPBS.

### 4.4. P-Selectin Binding/Adhesion Assay

To evaluate the P-selectin ligand expression on AsPC-1, Capan-2, and MIA PaCa-2 cells, respectively, 0.3 µg recombinant human P-selectin Fc Chimera Protein in 50 µL DPBS (R&D systems) was immobilized in each well of a Nunc MaxiSorp flat-bottom 96-well plate for 12 h at 4 °C. Afterwards, unspecific binding sites were blocked with 250 µL BSA solution (4% in DPBS) per well for 4 h and wells were rinsed with 250 µL DPBS thrice. Tumor cells were detached with EDTA (0.2 g/L EDTA × 4 Na, Sigma Aldrich) for 10 min at 37 °C, labeled with Calcein-AM (1 µM Calcein-AM concentration) for 1 h, and rinsed twice with warm DPBS buffer. Then, 5 × 10^5^ tumor cells in 100 µL DPBS were added in each well and the plate was gently shaken on a plate shaker for 2 h. Unbound cells were removed with DPBS and adherent cells were lysed with 1% Triton X-100 (*v/v*) in DPBS (Merck). Calcein-AM was quantified in a FLUOstar Optima plate reader (BMG Labtech, Ortenberg, Germany) at an excitation wavelength of 485 nm and an emission wavelength of 520 nm.

### 4.5. Light Transmission Aggregometry

Tumor cell-induced platelet aggregation was measured by light transmission aggregometry using an APACT-4004 aggregometer (Haemochrom Diagnostica, Essen, Germany) with platelets in buffer. Buffer was set as 100% and platelets in buffer as 0% light transmission. Platelets were prepared and co-incubated with different inhibitors as described in the previous section. Aggregate formation was induced by adding 2000 tumor cells to 200 µL platelets in buffer at 37 °C in adequate cuvettes to reach a final concentration of 1 × 10^4^ tumor cells/mL. Platelets and tumor cells were stirred continuously at 1000 rpm.

### 4.6. Platelet Dense Granule Secretion Assay

For quantification of ATP release from platelets dense granules, platelets in buffer were incubated with different inhibitors or DPBS for 30 min, respectively. AsPC-1, Capan-2, and MIA PaCa-2 cells were detached with EDTA and suspended in DPBS. After incubation of platelets (4 × 10^8^ Plts/mL) with 1 × 10^4^ tumor cells/mL for 20 min, ATP concentration was quantified by luminescence measurement using a luciferin-based ATP-Determination Kit (Thermo Fisher Scientific, Waltham, MA, USA) and a FLUOstar Optima plate reader (BMG Labtech).

### 4.7. Thrombin Generation Assay

Tumor cell-induced thrombin generation was evaluated using a fluorogenic thrombin generation assay. To eliminate the effects of the intrinsic coagulation system, platelet-rich-plasma was supplemented with Corn Trypsin Inhibitor (Santa Cruz Biotechnology). Tumor cells were added to a final concentration of 1 × 10^4^ cells/mL. After adding the fluorogenic substrate Technothrombin TGA SUB (Technoclone GmbH, Vienna, Austria) and recalcification, the kinetic of the subsequent thrombin mediated Z-Gly-Gly-Arg-AMC cleavage was measured immediately and fluorescence was converted into thrombin concentrations using the evaluation software provided by Technoclone GmbH.

### 4.8. Statistical Analysis

Comparisons were performed using the software Prism (GraphPad Software, San Diego, CA, USA). Student’s t test was used to compare two groups, and one-way analysis of variance (ANOVA) was used for three or more groups. *p* < 0.05 was considered statistically significant and marked with an asterisk. Two asterisks indicate a p-value below 0.05 and three stars were used for *p*-values below 0.001.

## Data Availability

Data is contained within the article and supplementary material.

## Abbreviations

PAR-1	Protease-activated Receptor-1
TF	Tissue Factor
TRAP-6	Thrombin Receptor Activator Peptide 6
TXA2	Thromboxane A2
CLEC-2	C-type lectin-like receptor-2
DMSO	Dimethyl Sulfoxide
PRP	Platelet-rich-plasma
EDTA	Ethylenediaminetetraacetic acid
Plts	Platelets
ATP	Adenosine triphosphate
NK-cell	Natural killer cell
MAP kinase	mitogen-activated protein kinase
GP	Glycoprotein
